# The role of caspase-1, caspase-4 and NLRP3 in regulating the host cell response evoked by uropathogenic *Escherichia coli*

**DOI:** 10.1038/s41598-022-06052-7

**Published:** 2022-02-07

**Authors:** Anna Lindblad, Charlotte Johansson, Katarina Persson, Isak Demirel

**Affiliations:** School of Medical Sciences, Inflammatory Response and Infection Susceptibility Centre (iRiSC), Örebro University, 701 82 Örebro, Sweden

**Keywords:** Cytokines, Immune evasion, Infection

## Abstract

The inflammasome-associated proteins caspase-1, caspase-4 and NLRP3 have been emphasised to be essential in the host cell response during urinary tract infection (UTI) by regulating IL-1β release. Our aim was to investigate how the inflammasome-associated proteins regulate the cell response of bladder epithelial cells during infection with uropathogenic *Escherichia coli* (UPEC). Human bladder epithelial cells (5637) and CRISPR/Cas9 generated caspase-1, caspase-4 and NLRP3 knockdown cells were stimulated with the UPEC strain CFT073. Using Olink proteomics and real time RT-PCR, we showed that caspase-1, caspase-4 and NLRP3 are vital for the expression of many inflammatory genes and proteins from bladder epithelial cells. When investigating the effect of inflammasome-associated proteins on neutrophils, we found that conditioned medium from UPEC-infected caspase-4 knockdown cells significantly increased phagocytosis of CFT073 and significantly decreased ROS production from neutrophils. In contrast, conditioned medium from UPEC-infected NLRP3 knockdown cells significantly decreased the phagocytosis of CFT073 and significantly increased the ROS production from neutrophils. In conclusion, we showed that the inflammasome-associated proteins contribute to the host cell response during UPEC infection.

## Introduction

Urinary tract infection (UTI) caused primarily by uropathogenic *Escherichia coli* (UPEC) is one of the most common infections in humans^1,2^. It is estimated that 50% of all women will experience at least one UTI during their lifetime. In addition, women with a history of UTI seem to have an increased risk of recurrency within 3 to 4 months^2^. UPEC have the ability to cause symptomatic UTI by utilizing virulence factors such as type-1 fimbriae and P-fimbriae, capsule, lipopolysaccharides (LPS), α-hemolysin, iron acquisition systems, SisA and SisB^1,3^. To successfully colonize bladder epithelial cells, UPEC have developed a wide range of mechanisms to avoid the innate immune system. They can suppress epithelial cytokine production by controlling NF-κB activity by genes involved in LPS biosynthesis or in α-hemolysin (HlyA)-dependent way^4^. The UPEC strain CFT073 has been shown to inhibit TLR signaling by the virulence factor TcpC in a MyD88 dependent way and also TRIF and IL-6/IL-1 signaling cascades independent of MyD88^4^. UPEC can also adhere to bladder epithelial cells by type-1 fimbriae-dependent mechanisms and invade. The vast majority of the invading UPEC will be expelled back into the bladder lumen following exocytosis^5^. However, UPEC that are not being expelled from the cells may establish intracellular reservoirs called intracellular bacterial communities (IBC)^6,7^.

Inflammasomes are multiprotein complexes that evoke an immune response by the induction of pyroptosis and maturation and secretion of proinflammatory cytokines such as IL-1β and IL-18. The formation of a inflammasome is initiated by pattern recognition receptors (PRRs) which sense pathogen-associated molecular patterns (PAMPs) and danger-associated molecular patterns (DAMPs)^8^. When the inflammasome assembles, it activates different caspases, which are proteases that are found in an inactive pro-form in the cytosol and are activated by autoproteolytic cleavage or by other caspases^9,10^. Caspase-1 and caspase-4 are classified as proinflammatory caspases and they can induce pyroptosis in response to different stimuli. Upon activation, the proinflammatory caspases cleave and activate the pore-forming protein gasdermin D which is required for pyroptosis mediated cell death^8,11^. Pyroptosis can be measured by the release of lactate dehydrogenase (LDH) from dying cells in combination with the activation of proinflammatory caspases. During canonical inflammasome activation, caspase-1 converts pro-IL-1β into active IL-1β and mediates the release of the cytokine^8^. The best studied inflammasome is the NACHT leucin-rich repeat PYD protein 3 (NLRP3) inflammasome. Also, activation of human caspase-4/-5 and the mouse homologue caspase-11 by different Gram-negative bacteria leads to non-canonical inflammasome activation and pyroptosis. It has been shown that caspase-11 initiates NLRP3 and ASC-dependent activation of caspase-1 and maturation of pro-IL-1β^12^. Human caspase-4 and mouse caspase-11 were reported to be cytosolic LPS receptors leading to non-canonical inflammasome activation independently of TLR4^12,13^. Recently, caspase-4 was found to induce inflammasome mediated IL-1β release and pyroptosis by binding to LPS and disaggregating large LPS micelles into smaller LPS/caspase-4 complexes^11^. The knowledge that caspase-4 is a cytosolic LPS receptor is interesting and bring to light a possible role for inflammasome-associated proteins in establishment of intracellular bacteria. It is not known today if caspase-4 can sense UPEC LPS and regulate the intracellular persistence of UPEC in bladder epithelial cells e.g., through pyroptosis. It has been emphasized that the NLRP3 inflammasome plays an important role during a UPEC mediated UTI^14^. However, the data is contradictory. One study has shown that mice infected with UPEC induced IL-1β release and that IL-1β was contributing to the severity of the infection. Given that mice lacking IL-1β were protected from developing UTI^15^. However, others have reported that UPEC-induced IL-1β is part of the immune response that is limiting the colonization of the urinary tract^16,17^. It has recently been emphasized that inflammasome-associated proteins are not only important for the release of IL-1β and IL-18, but they also function as transcription regulators^15^. There is at present limited knowledge regarding the contribution of caspase-1, caspase-4 and NLRP3 in the release of other inflammatory mediators from the bladder epithelial cells upon a UPEC infection. The aim of this study was to investigate how the inflammasome-associated proteins caspase-1, caspase-4 and NLRP3 regulate the cell response of bladder epithelial cells during UPEC infection.

## Material and methods

### Human bladder epithelial cells

The human bladder epithelial cell line 5637 (ATTCC HBT-9), obtained from the American Type Culture Collection (Manassas, VA, USA) were cultured in Dulbecco’s modified Eagle Medium (DMEM) (BioWhittaker, Lonza, Switzerland) supplemented with 10% fetal bovine serum (FBS), 1 mM non-essential amino acids and 2 mM L-glutamine (Hyclone, GE Lifescience, UK) at 37 °C with 5% CO_2_. During infection experiments, cell culture medium was substituted with DMEM containing 2% FBS, 1 mM non-essential amino acids and 2 mM L-glutamine, as previously described^18^.

### CRISPR/Cas9 genome editing

The pSpCas9 (BB)-2A-Puro (PX459, V2.0) (a gift from Feng Zhang, Addgene plasmid # 62988)^19^ was used for CRISPR/Cas9 gene editing in bladder epithelial cells using Lipofectamine 2000 (Life Technologies, Carslbad, CA, USA). The target sites were: GCTAATGATCGACTTCAATG (NLRP3), TGCAGCTCATCCGAATATGG (Caspase-4) and GACAGTATTCCTAGAAGAAC (Caspase-1). The cells were selected by using pyromycin (2.5 µg/ml, Sigma-Aldrich, St. Louis, MO, USA) 24 h after transfection. All experiments were conducted with a polyclonal pool of gene-edited cells. The phenotype was confirmed by Western Blot analysis, as previously described^18^.

### Bacterial strains and growth conditions

The UPEC strain CFT073 originally isolated from a patient with acute pyelonephritis^20^ and grown on tryptic-soy agar (TSA) (Becton Dickison, Le Pont Claix, France). Prior to experiments, bacteria were inoculated in Difco Luria–Bertani (LB) broth (Lennox; Franklin Lakes, NJ, USA) and incubated at 37 °C aerobically on a shaker overnight, as previously described^18^.

### Stimulation of bladder epithelial cells

**Figure 1:**
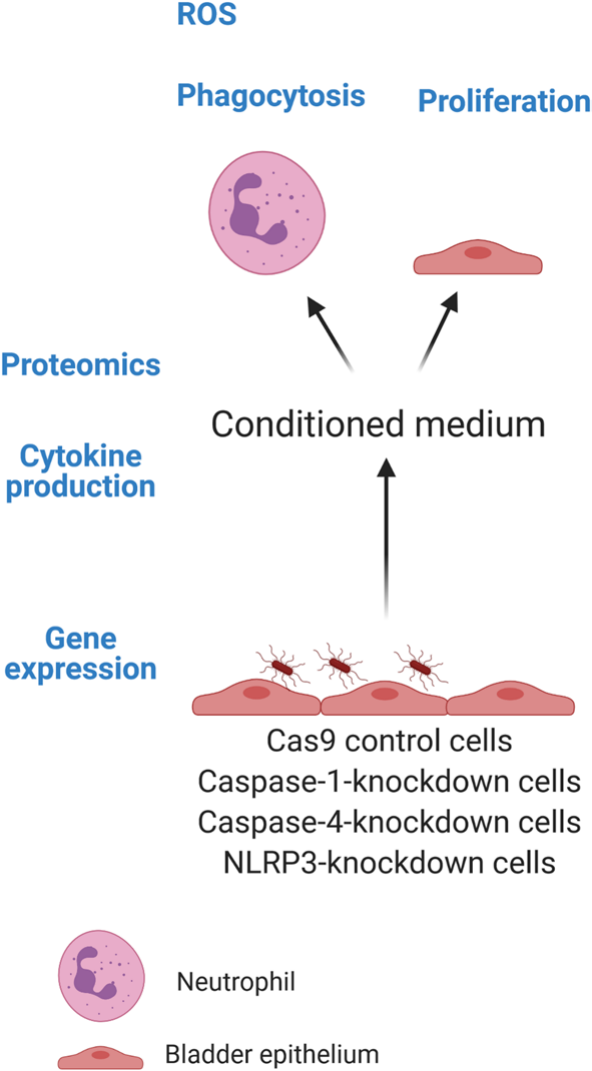
A schematic overview summarizing the experimental design. Caspase-1, caspase-4 and NLRP3-knockdown bladder epithelial cells and Cas9 controls were infected with the UPEC strain CFT073. Measurement of gene expression and cytokine release (IL-1β, IL-18, IL-8) from infected cells was evaluated by ELISA. Conditioned medium collected from respective knockdown cell line was analyzed by proteomics using a panel of inflammatory proteins. The conditioned medium was also used to evaluate ROS production and phagocytosis in neutrophils and cell proliferation from bladder epithelial cells (created with Biorender.com).

The bladder epithelial cell line 5637 (Cas9 controls, caspase-1, caspase-4 and NLRP3-knockdown cells) were stimulated with CFT073 for 6 h at a multiplicity of infection (MOI) of 10 and incubated at 37 °C with 5% CO_2_. Supernatants were collected and kept at − 80 °C until analysis, as previously described^18^. A schematic overview of the experimental design of the study is shown in Fig. 1.

### Measurement of cytokine release and cell viability

An enzyme-linked immunosorbent assay (ELISA) was performed to measure IL-1β, IL-8 (ELISA MAX Deluxe Sets, (BioLegend, San Diego, USA) and IL-18 (Duo set, ELISA, R&D Systems, Minneapolis, USA) from bladder epithelial cells according to the manufacturer’s instructions. A lactate dehydrogenase (LDH) assay (CyQUANT LDH Cytotoxicity Assay Kit, Thermo fisher Scientific, Massachusetts, USA) was performed to measure cell viability according to the manufacturer’s instructions. Both assays were analysed using the Cytation 3 plate reader (BioTek, Winooski, VT, USA), as previously described^18^.

### RNA isolation and real time RT-PCR

Total RNA isolation from bladder epithelial cells was performed by using E.Z.N.A. Total RNA Kit I (Omega Bio-tek, GA, USA) according to manufacturer’s instructions. The quantity of RNA was determined using spectrophotometry (Nano-Drop ND-1000, Wilmington, NC, USA). First strand cDNA synthesis was performed by High-capacity cDNA RT kit (Thermo Fisher Scientific). The real time-RT-PCR was conducted with Maxima SYBR Green qPCR Master Mix (Thermofisher), with 250 nM of each primer (Supplementary Table S1) designed by Origene (Maryland, USA) and synthesised by Eurofins MWG Synthesis GmbH (Ebersberg, Munich)) and 10 ng cDNA. The PCR amplification was performed in a CFX96 Touch Real-Time PCR Detection System (Bio-Rad Laboratories, Hercules, CA, USA) using the following protocol: initial denaturation at 95 °C for 10 min, 40 cycles of denaturation at 95 °C for 15 s followed by annealing/extension at 60 °C for 60 s. The CT-values were analyzed by the comparative Ct (ΔΔCt) method and normalized to the endogenous control GAPDH. Fold difference was calculated as 2^−ΔΔCt^^21^.

### Western blot analysis

The bladder epithelial cells were lysed in radioimmunoprecipitation assay (RIPA) buffer supplemented with a phosphatase inhibitor mix (Thermo Fisher Scientific). The DC protein assay (Bio-Rad Laboratories, Hercules, CA, USA) was used for protein quantification. The proteins were mixed with equal amounts of Laemmli buffer, boiled for 5 min in 95 °C and separated on a 4–20% SDS–polyacrylamide gel electrophoresis and transferred onto a polyvinylidene fluoride membrane (PVDF) (Bio-Rad Laboratories). The PVDF membrane was blocked with 3% BSA in Tris-buffered Saline 0.1% Tween 20 (TBST) for 1 h at room temperature and incubated over night at 4 °C with the primary antibodies. Human caspase-1 was detected using a mouse monoclonal antibody (AdipoGen Life Sciences, Buckingham, UK). Human caspase-4 was detected using a rabbit monoclonal antibody (Cell signaling Technologies, Massachusetts, USA) and human NLRP3 was detected by using a mouse monoclonal antibody (Abcam, Cambridge, UK). GAPDH was detected by using a rabbit polyclonal antibody (Santa Cruz Biotechnology, Dallas, Texas, USA). As secondary antibodies, goat anti-rabbit IgG (HRP) (Abcam, Cambridge, UK) and goat anti-mouse IgG (HRP) (Abcam, Cambridge, UK) were used and incubated for 1 h at room temperature. The bands were imaged using Luminata Forte Western HRP Substrate (Merck Millipore, Darmstadt, Germany), as previously described^18^.

### Neutrophil isolation

Human neutrophils were isolated from healthy blood donors using density gradient centrifugation of polymorphprep and lymphoprep reagents (AXIS-SHIELD PoC AS, Oslo, Norway) according to the manufacture’s instruction. The regional ethics review board in Uppsala, Sweden has given their approval (Dnr 2015/437) to isolate blood from healthy individuals after informed consent. The collection of blood from healthy donors were performed according to the ethical guidelines of both the Declaration of Helsinki and the Swedish national board of health and welfare. The viability of neutrophils was examined by microscopy, as previously described^22^.

### Conditioned medium

The Cas9, caspase-1, caspase-4 and NLRP3-knockdown cells were stimulated with CFT073 for 6 h at MOI 10 and incubated at 37 °C with 5% CO_2_. Supernatants were collected and centrifuged at 5000 g for 5 min to remove the bacteria and then frozen at − 80 °C until used. These supernatants were defined as conditioned medium. No presence of viable bacteria was found in the conditioned medium after plating on TSA agar.

### Measurement of reactive oxygen species (ROS)

To measure total reactive oxygen species (ROS) production from neutrophils a luminol-horseradish peroxidase (HRP) assay was performed. The neutrophils were incubated with luminol (0.1 mg/ml, Sigma) and HRP (4 U/ml, Roche) for 15 min at 5% CO_2_ in 37 °C. The neutrophils (10^6^) were added to a 96-well plate with respective conditioned medium from the infected bladder epithelial cells (cas9, caspase-1, caspase-4 and NLRP3). The luminescence was measured in a microplate reader (Cytation 3) every third min for 3 h, as previously described^22^.

### Phagocytosis assay

Conditioned medium was transferred to a 96-well plate and neutrophils (200,000 cells) were added to respective well. CFT073 (carrying an eGFP-plasmid) at MOI 10 was added to the wells containing conditioned medium and neutrophils for 3 h. The neutrophils were washed twice with PBS to remove non-phagocytosed bacteria and then 0.2% Trypan blue (Thermo fisher Scientific) was added to quench the remaining eGFP signal from extracellular bacteria. Phagocytosis was quantified by measuring the mean florescence intensity of the phagocytized CFT073 (eGFP) bacteria using the Gallius (Beckman Coulter, Brea, USA) flow cytometer with 488 nm laser and FL1 525/40 nm band-pass filter. The data was analyzed with Kaluza Flow Cytometry Analysis v1.3 (Beckman Coulter), as previously described^22^.

### Proliferation assay

Conditioned medium was transferred to a 96-well plate with wild-type 5637 cells (50% confluent) and incubated for 24 h at 5% CO_2_ in 37 °C. After 24 h, the 5637 cells were washed once with PBS and 0.1% crystal violet (Sigma-Aldrich) was added to the cells for 10 min and then washed twice with tap water. Cells were then destained with 1% sodium dodecyl sulfate on a shaker at 500 rpm for 5 min. The optic density was measured with a plate reader (Cytation 3) at 570 nm. After 24 h of stimulation, the cells were also counted using a Bürker counting chamber.

### Targeted protein analysis

Bladder epithelial cells were stimulated with CFT073 at MOI 10 for 6 h. Cell supernatants were collected and centrifuged at 5000 g for 5 min and stored at – 80 °C. A panel of 92 inflammatory proteins were analysed in the supernatants by using the proximity extension assay (PEA) technology (Olink Bioscience AB, Uppsala, Sweden). The protein data are reported as normalized protein expression levels (NPX). Proteins that expressed signals below the limit of detection (LOD) were excluded from further analysis, as previously described^18^.

### Statistical methods

Data are presented as mean ± standard error of the mean (SEM). Differences between groups were evaluated by unpaired Student’s t-test or one-way ANOVA followed by Bonferroni multiple testing correction. Results were considered statistically significant at *p* < 0.05. n = number of independent experiments.

## Results

### Involvement of inflammasome-associated proteins in IL-1β release

**Figure 2:**
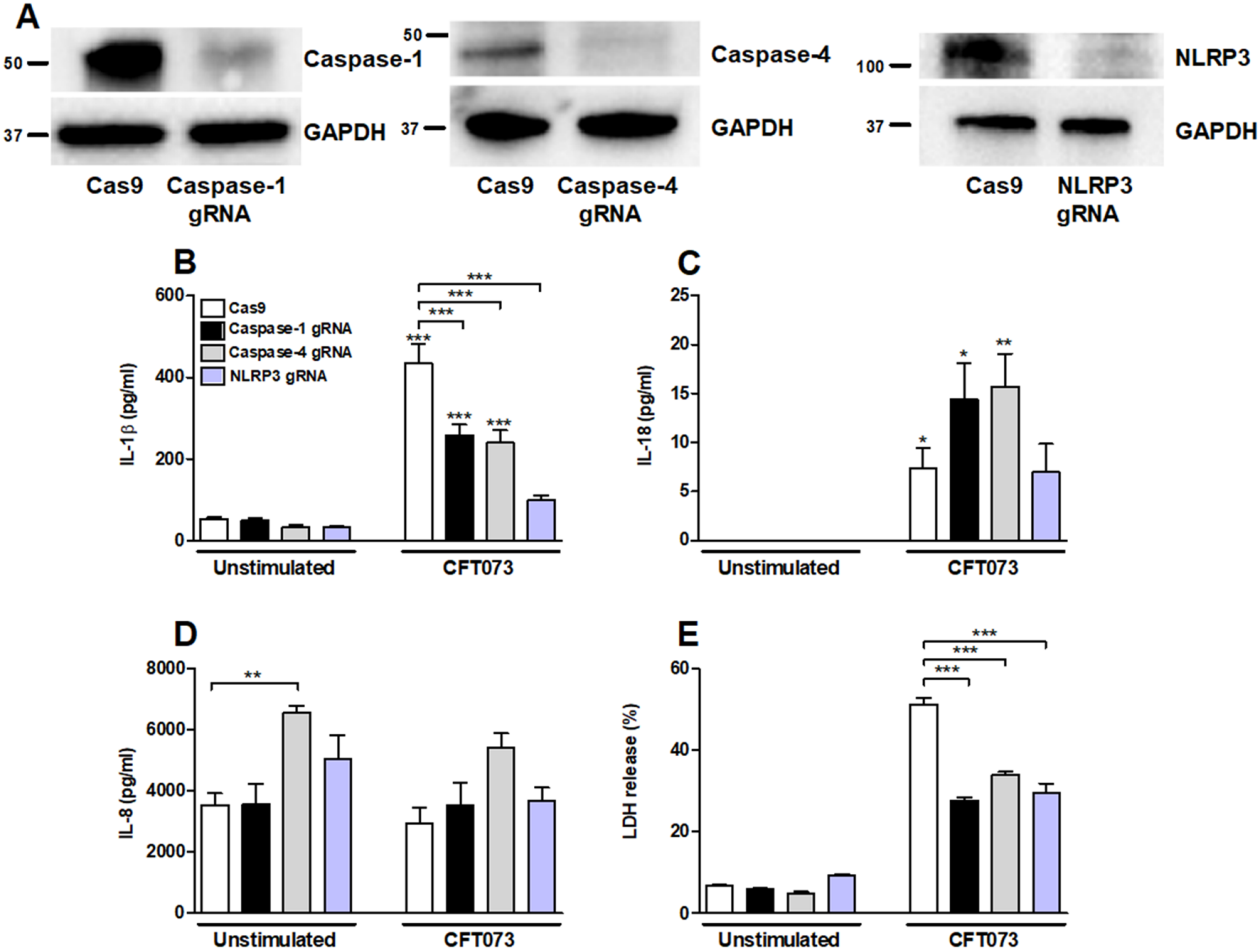
The knockdown of caspase-1, caspase-4 and NLRP3 in bladder epithelial cells using the CRISPR/Cas9 system was assessed by Western blot analysis. GAPDH was used as loading control (**A**). Original western blot presented is available in Supplemental Fig. S1. IL-1β (**B**), IL-18 (**C**), IL-8 (**D**) and LDH (**E**) release from Cas9, caspase-1, caspase-4 or NLRP3-knockdown bladder epithelial cells after stimulation with UPEC strain CFT073 at MOI 10 for 6 h. gRNA stands for guideRNA targeting specific gene using CRISPR/Cas9. Data are presented as mean ± SEM of three independent experiments. Asterisks over error-bar show statistical significance compared to respective unstimulated control (*p < 0.05, **p < 0.01, ***p < 0.001).

Bladder epithelial cells knockdown in caspase-1, caspase-4 and NLRP3 were constructed with the CRISPR/Cas9 system. Western blot analysis confirmed a decreased protein expression (knockdown efficiency) of caspase-1 (81%), caspase-4 (70%) and NLRP3 (76%) (Fig. 2A) compared to bladder epithelial cells transfected with an empty control Cas9 plasmid. The knockdown cells were then stimulated with the UPEC strain CFT073 at MOI 10 for 6 h and the IL-1β release was evaluated. When comparing the stimulated cells to their own unstimulated control, all but NLRP3-knockdown cells showed a significant increased release of IL-1β (Fig. 2B). Caspase-1, caspase-4 and NLRP3-knockdown cells released significantly lower IL-1β upon CFT073 stimulation compared to the CFT073 stimulated Cas9 control cells (Fig. 2B). We also found that CFT073 induced a significantly lower LDH release (Fig. 2E) from caspase-1, caspase-4 and NLRP3-knockdown cells compared to Cas9 control cells. Taken together, these results show that caspase-1, caspase-4 and NLRP3 are involved in IL-1β and LDH release from bladder epithelial cells.

### Involvement of inflammasome-associated proteins in IL-18 and IL-8 release

We continued with investigating the involvement of inflammasome-associated proteins in UPEC-evoked release of IL-18 and IL-8 from bladder epithelial cells. We found that Cas9, caspase-1, caspase-4, but not NLRP3-knockdown cells were able to induce a significantly increased IL-18 release compared to unstimulated cells (Fig. 2C). However, the release of IL-8 was not significantly different in caspase-1, caspase-4 or NLRP3-knockdown cells infected with CFT073 compared to unstimulated control cells (Fig. 2D). Caspase-4-knockdown cells induced a significantly increased basal release of IL-8 compared to unstimulated Cas9 cells (Fig. 2D).

### mRNA expression of inflammation-related genes

**Figure 3:**
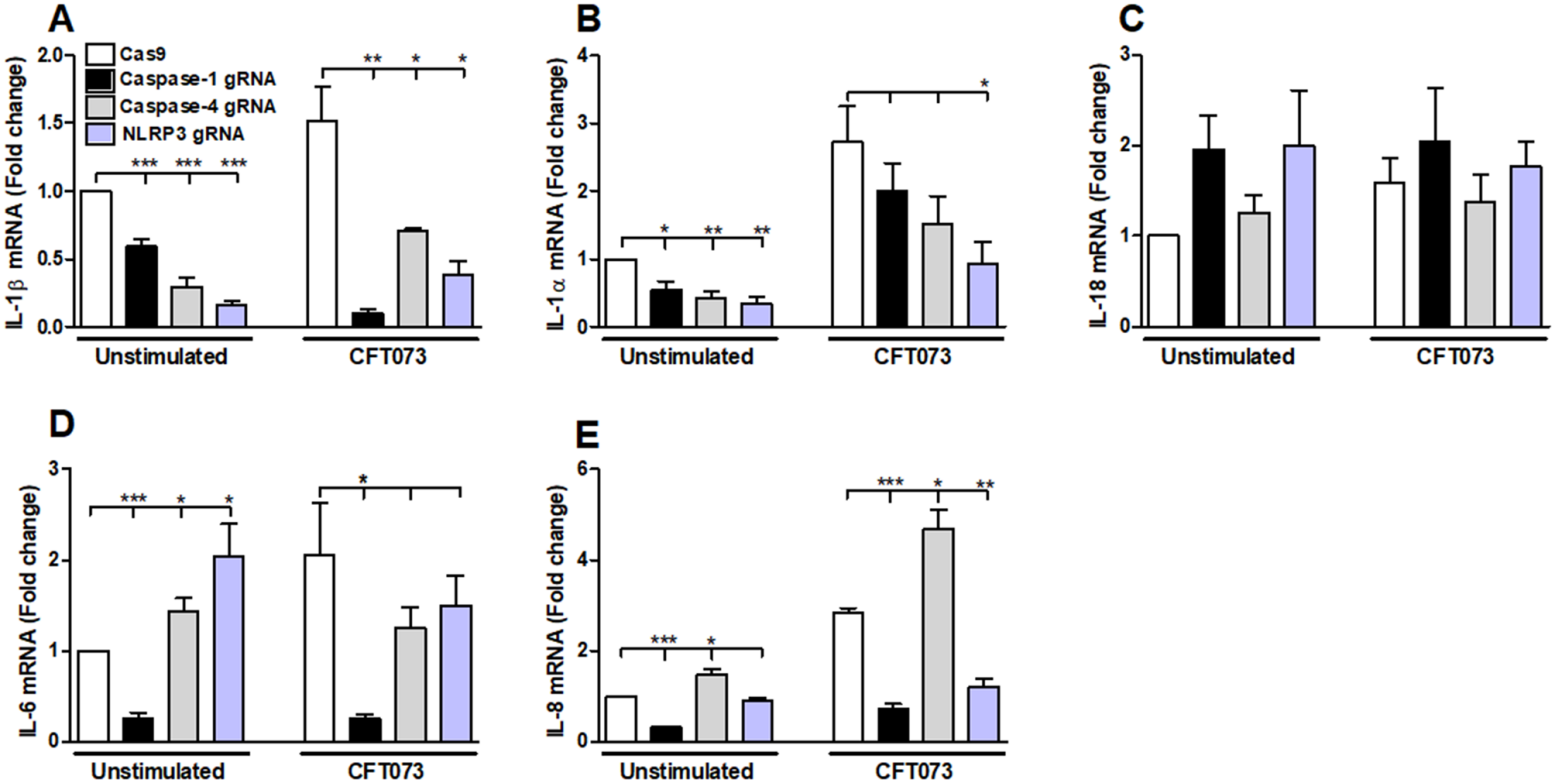
Gene expression of inflammasome-related genes. Caspase-1, caspase-4 and NLRP3-knockdown bladder epithelial cells and Cas9 controls were infected with CFT073 followed by analysis of mRNA expression of IL-1β (**A**), IL-1α (**B**), IL-18 (**C**), IL-6 (**D**) and IL-8 (**E**). The gene expression was normalized to GAPDH and expressed as fold change relative to unstimulated controls. gRNA stands for guideRNA targeting specific gene using CRISPR/Cas9. Data are presented as mean ± SEM of three independent experiments. Asterisks denote statistical significance compared to Cas9 control cells (*p < 0.05, **p < 0.01, ***p < 0.001).

To investigate the expression of inflammation-related genes, bladder epithelial cells were stimulated with CFT073 for 6 h and the mRNA expression of IL-1β, IL-1α, IL-18, IL-6 and IL-8 was measured. We found that cells stimulated with CFT073 showed a significant decreased mRNA expression of IL-1β in caspase-1, caspase-4 or NLRP3-knockdown cells compared to CFT073 stimulated Cas9 control cells (Fig. 3A). The basal mRNA expression of IL-1β and IL-1α was significantly reduced in caspase-1, caspase-4 or NLRP3-knockdown cells compared to unstimulated Cas9 cells. Only NLRP3-knockdown cells showed a significantly decreased mRNA expression of IL-1α after CFT073 stimulation (Fig. 3B). Furthermore, the mRNA expression of IL-6 was significantly decreased in caspase-1-knockdown cells (Fig. 3D). In addition, caspase-4 and NLRP3-knockdown cells showed an increased expression of basal IL-6 expression (Fig. 3D). CFT073 stimulated caspase-1 and NLRP3-knockdown cells induced a decreased mRNA expression of IL-8 (Fig. 3E). However, no differences in the mRNA expression of IL-18 were noted in caspase-1, caspase-4 or NLRP3-knockdown cells (Fig. 3C).

### Proteomics

**Figure 4:**
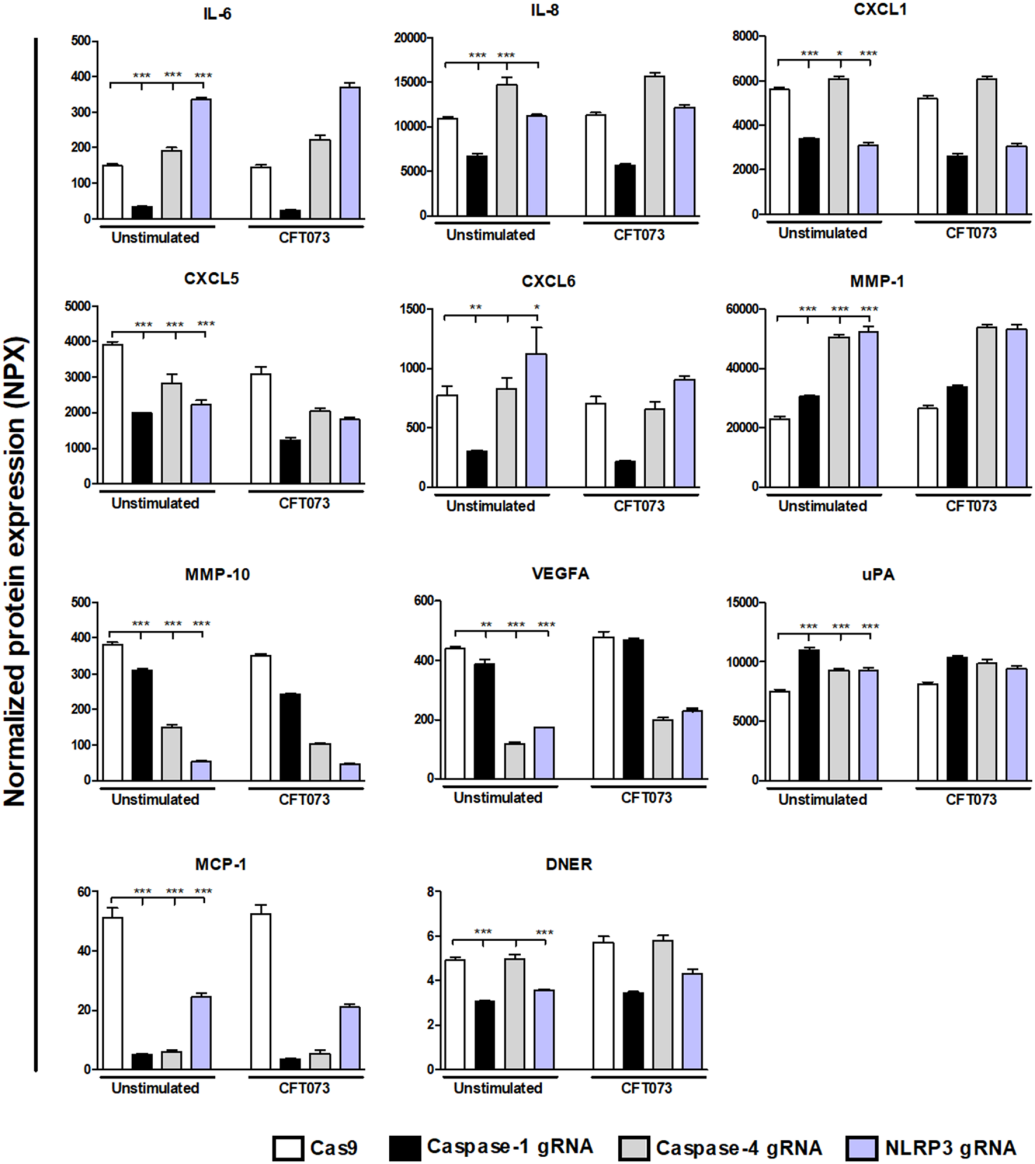
Targeted protein analysis of basal protein expression from bladder epithelial cells knockdown in caspase-1, caspase-4 or NLPR3. Caspase-1, caspase-4 and NLRP3-knockdown bladder epithelial cells and Cas9 controls were infected with CFT073 followed by analysis of protein expression using a panel of inflammation-related proteins. The basal protein expression was either significantly increased or decreased compared to Cas9 controls. The protein data is presented as normalized protein expression (NPX). gRNA stands for guideRNA targeting specific gene using CRISPR/Cas9. Data are presented as mean ± SEM of three independent experiments. Asterisks show statistical significance compared to Cas9 controls (*p < 0.05, **p < 0.01, ***p < 0.001).

**Figure 5:**
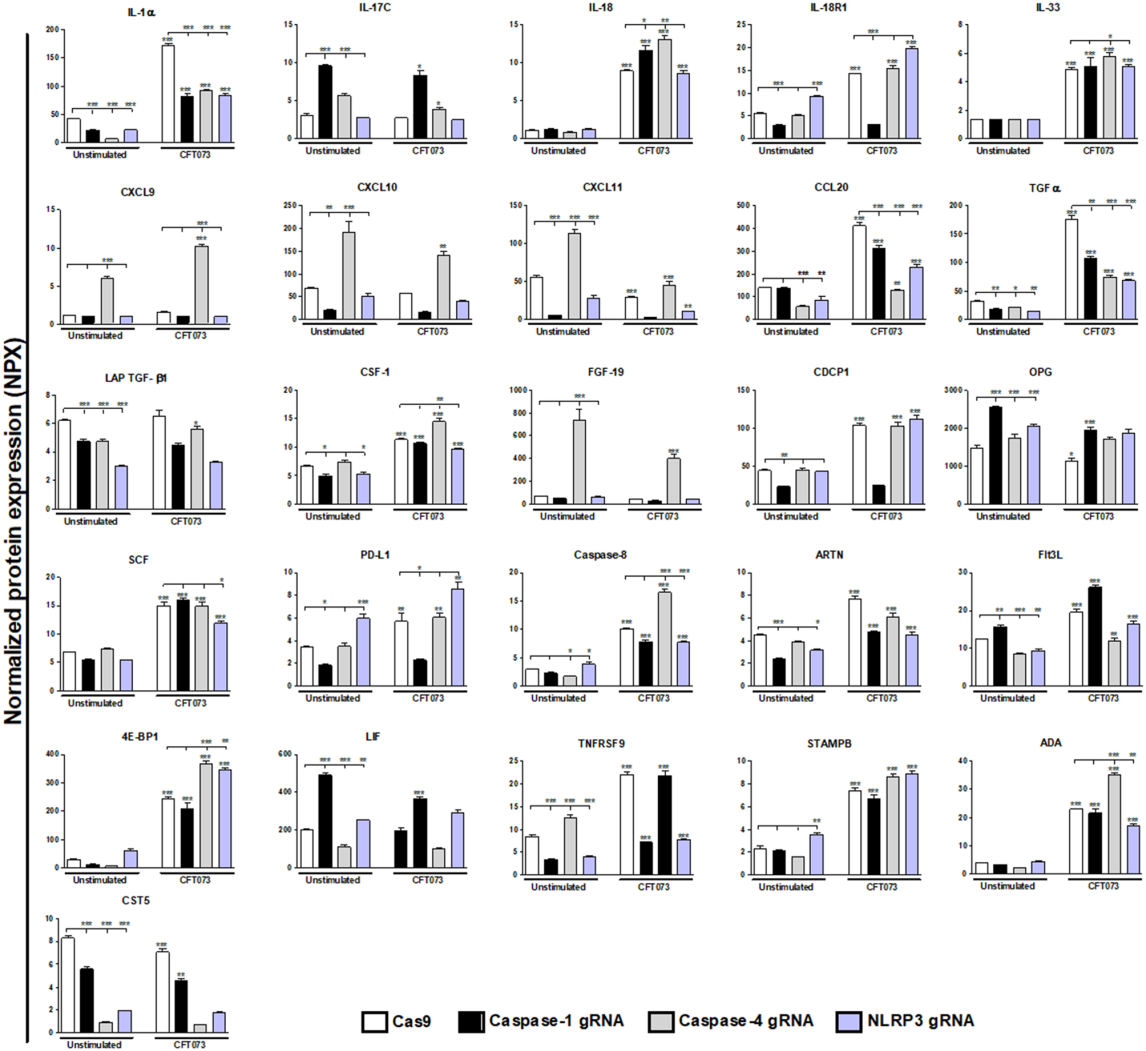
Targeted protein analysis of basal and UPEC-induced protein expression from bladder epithelial cells knockdown in caspase-1, caspase-4 or NLPR3. Caspase-1, caspase-4 and NLRP3-knockdown bladder epithelial cells and Cas9 controls were infected with CFT073 followed by analysis of protein expression using a panel of inflammation-related proteins. All proteins that responded to stimulation with the UPEC strain CFT073 with either a significant increase or decrease in protein expression are presented. The protein data is presented as normalized protein expression (NPX). gRNA stands for guideRNA targeting specific gene using CRISPR/Cas9. Data are presented as mean ± SEM of three independent experiments. Asterisks over error-bar show statistical significance compared to respective unstimulated control (*p < 0.05, **p < 0.01, ***p < 0.001).

To gain further knowledge about the interaction of caspase-1, caspase-4 and NLRP3 with other host response factors during UPEC infection, an Olink proteomics analysis were conducted of 92-inflammatory proteins (Supplementary Table S2). A total of 32 proteins showed a significantly changed basal protein expression compared to Cas9 control cells after 6 h (Figs. 4, 5). Caspase-1-knockdown cells showed significantly decreased expression in 21 proteins (IL-6, IL-8, CXCL1, CXCL5, CXCL6, CXCL10, CXCL11, MMP-10, VEGFA, MCP-1, DNER, IL-1α, IL-18R1, TGF-α, LAP-TGF-β1, CSF-1, CDCP1, PD-L1, ARTN, TNFRSF9 and CST5) and increased expression in 6 proteins (MMP-1, uPA, IL17C, OPG, FIt3L and LIF). Caspase-4-knockdown cells showed significantly decreased expression in 12 proteins (CXCL5, MMP-10, VEGFA, MCP-1, IL-1α, CCL20, TGF-α, LAP-TGF-β1, Caspase-8, Flt3L, LIF, CST5) and increased expression in 12 proteins (IL-6, IL-8, CXCL1, CXCL9, CXCL10, CXCL11, MMP-1, uPA, IL-17C, FGF-19, OPG and TNFRSF9). NLRP3- knockdown cells showed significantly decreased expression in 16 proteins (CXCL1, CXCL5, CXCL11, MMP-10, VEGFA, MCP-1, DNER, IL-1α, CCL20, TGF-α, LAP-TGF-β1, CSF-1, ARTN, Flt3L, TNFRSF9 and CST5) and increased expression in 10 proteins (IL-6, CXCL6, MMP-1, uPA, IL-18R1, OPG, PD-L1, Caspase-8, LIF and STAMPB).

Next, we explored how the CFT073 infection altered the protein release from the knockdown cells independently of basal changes. We found 26 proteins that were significantly altered by the CFT073 infection in caspase-1, caspase-4 or NLRP3-knockdown cells compared to CFT073 stimulated Cas9 control cells or compared to their own unstimulated controls (Fig. 5). Three proteins (IL-1α, TGFα and CCL20) were significantly decreased in CFT073-stimulated caspase-1, caspase-4 and NLRP3-knockdown cells compared to CFT073 stimulated Cas9 control cells. When comparing CFT073-stimulated caspase-1-knockdown cells with CFT073 stimulated Cas9 control cells, only one significantly increased protein was found (IL-18). However, five proteins (IL-18R1, IL-1α, PDL-1, TGFα and CCL20) were significantly decreased in CFT073 stimulated caspase-1-knockdown cells. When comparing CFT073-stimulated caspase-4-knockdown cells with CFT073-stimulated Cas9 cells, three proteins (TGFα, IL-1α and CCL20) showed a significant decrease and seven proteins (IL-18, IL-33, 4E-BP1, Caspase-8, CSF-1, ADA and CXCL9) showed a significant increase. A total of 6 proteins (IL-1α, CCL20, TGFα, SCF, caspase-8 and ADA) were significantly decreased in CFT073-stimulated NLRP3-knockdown cells and one protein (4E-BP1) were significantly increased compared to CFT073-stimulated Cas9 cells.

### Phagocytosis and ROS-production in neutrophils induced by conditioned medium

**Figure 6:**
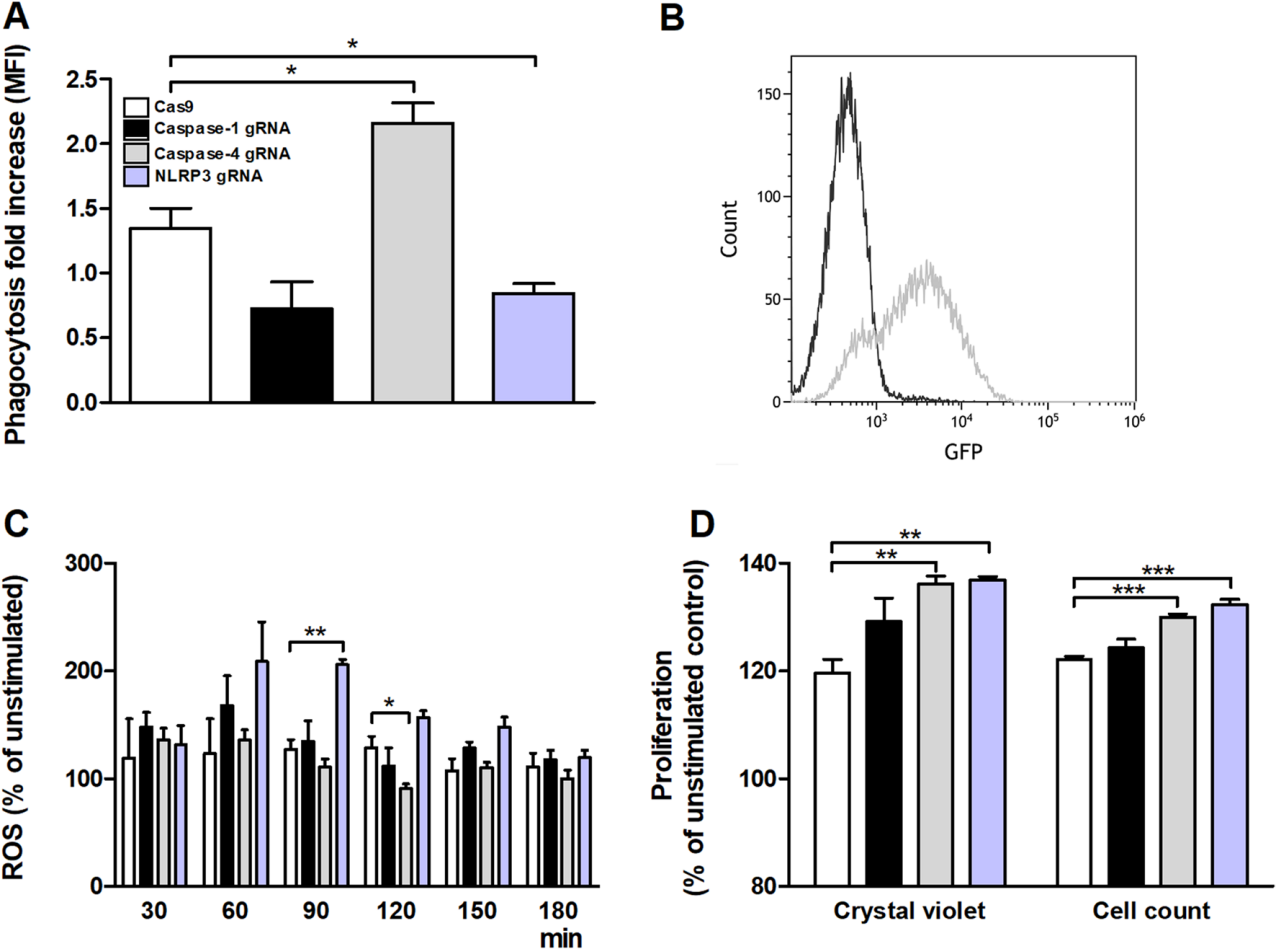
Bacterial phagocytosis, production of reactive oxygen species (ROS) and bladder epithelial cell proliferation. Neutrophils were exposed to conditioned medium from Cas9 controls, caspase-1, caspase-4 and NLRP3-knockdown bladder epithelial cells and phagocytosis of CFT073 (eGFP) (3 h, **A**, **B**) and ROS-production was evaluated (**C**). Conditioned medium from respective knockdown cell lines was added to wild-type epithelial cells and proliferation was then evaluated after 24 h (**D**). Phagocytosis is expressed as fold change (mean florescence intensity) relative to the unstimulated control. A representative flow cytometry histogram illustrating neutrophils with (grey) or without (black) phagocytosis of CFT073 is shown in B. ROS production is presented as % of unstimulated control. gRNA stands for guideRNA targeting specific gene using CRISPR/Cas9. Data are presented as mean ± SEM of three independent experiments. (*p < 0.05, **p < 0.01, ***p < 0.001).

The proteomics analysis demonstrated that the expression of many inflammatory proteins in conditioned medium harvested from UPEC-infected bladder epithelial cells with a deficiency in caspase-1, caspase-4 and NLRP3 was different from control cells. We continued to investigate how conditioned medium collected from the caspase-1, caspase-4 and NLRP3-knockdown cells influenced phagocytosis and ROS-production by neutrophils. Neutrophils were exposed to conditioned medium from CFT073 stimulated Cas9, caspase-1, caspase-4 and NLRP3-knockdown cells and phagocytosis and ROS-production was evaluated. We found that conditioned medium from caspase-4-knockdown cells induced an increased phagocytosis of CFT073, while conditioned medium from NLRP3-knockdown cells induced decreased phagocytosis of CFT073 compared to Cas9 control cells. No significant alterations could be seen for caspase-1-knockdown cells (Fig. 6A,B). Upon examining ROS-production, conditioned medium from CFT073 stimulated NLRP3-knockdown cells induced a significant increased ROS-production in neutrophils compared to conditioned medium from CFT073 stimulated Cas9 control cells after 90 min (Fig. 6C). However, conditioned medium from CFT073 stimulated caspase-4-knockdown cells induced a significant decreased ROS-production in neutrophils compared to conditioned medium from CFT073 stimulated Cas9 control cells after 120 min (Fig. 6C). No alteration in production could be seen for caspase-1-knockdown cells.

### Proliferation of bladder epithelial cells induced by conditioned medium

Next, we investigated how conditioned medium from CFT073 stimulated Cas9, caspase-1, caspase-4 and NLRP3-knockdown cells affected the proliferation of wild-type 5637 bladder epithelial cells. Conditioned medium collected from caspase-4 and NLRP3-knockdown cells, but not from caspase-1-knockdown cells, induced an increased proliferation of wild-type bladder epithelial cells, both when evaluated with crystal violet and with cell counting, compared to conditioned medium from Cas9 controls (Fig. 6D).

## Discussion

The inflammasome-associated proteins caspase-1, caspase-4 and NLRP3 have been emphasised to be vital in the host response during urinary tract infection by regulating IL-1β release^14,16,23^. In this study we showed that the release of IL-1β and LDH was significantly reduced in caspase-1, caspase-4 and NLRP3-knockdown cells compared to CFT073 stimulated Cas9 cells. This is in agreement with previous findings^21^, which strengthens the notion that IL-1β and LDH released from bladder epithelial cells in vitro is caspase-1, caspase-4 and NLRP3-dependent. It is known that caspase-1, which is the functional component of the NLRP3 inflammasome, cleaves pro-IL-1β to its active form^8^. This, in addition with the almost complete inhibited IL-1β release from CFT073 stimulated NLRP3-knockdown cells, indicates that NLRP3 could be the most vital factor for IL-1β secretion from bladder epithelial cells. In addition, we have previously shown that the serine protease mesotrypsin is involved in the release of pro-IL-1β from bladder epithelial cells infected with CFT073^21^. This might explain why the caspase-1 and caspase-4-knockdown cells still have the ability to release significant amount of IL-1β. Our data also showed that caspase-1, caspase-4 and NLRP3 are important for gene expression of IL-1β in bladder epithelial cells. Interestingly, caspase-4 which is a proteolytical enzyme and also function as a cytosolic LPS receptor^11^ was also found to be involved in the release of IL-1β and LDH. However, LPS stimulation per se did not induce any IL-1β release from bladder epithelial cells after 6 or 24 h (data not shown). Furthermore, we have previously shown that the UPEC mediated IL-1β release from bladder epithelial cells is α-hemolysin, p38, ERK1/2 and ROS dependant^21^.

The release of inflammation-related proteins from caspase-1, caspase-4 and NLRP3-knockdown cells was analysed using a multiplex assay including 92 key proteins. Results acquired from the protein analysis revealed co-regulation of caspase-1, caspase-4 and NLRP3 with several inflammatory proteins in bladder epithelial cells. At the basal level we found that caspase-1, caspase-4 and NLRP3-knockdown cells altered the release of several cytokines and chemokines. The basal protein release and the mRNA expression of IL-6 and IL-8 were significantly downregulated in caspase-1-knockdown cells but upregulated in caspase-4-knockdown cells. A basal increase of IL-8 from caspase-4-knockdown cells was also seen with ELISA and RT-qPCR. IL-8 is known to be an important pro-inflammatory mediator vital for the clearance of UPEC^24,25^. The urothelial cells rapidly start to secrete IL-8 upon a UPEC infection, and the chemokine is associated with recruitment of neutrophils to the infection site^26^. Hence, our findings suggest that caspase-1, caspase-4 and NLRP3 have a broader role than previously known, in regulating the basal expression of cytokines and chemokines in bladder epithelial cells. Furthermore, UPEC-induced alteration of inflammatory proteins showed that CFT073 could significantly increase the expression of IL-1α, IL-18 and IL-33, members of the IL-1 superfamily. IL-1 has been associated with the host response during UPEC mediated infection, but IL-18 and IL-33 currently lack a known role in the host response during UPEC infection. Interestingly, in caspase-1-knockdown cells IL-18 was increased upon CFT073 infection, but the expression of the IL-18 receptor was reduced to basal levels. This could be due to the pharmacodynamic phenomenon that the receptor population usually downregulates as an adaption to higher availability of agonists. We also found that the release of IL-1α and IL-1β was reduced from caspase-1, caspase-4 and NLRP3-knockdown cells, in agreement with previous findings^23^. Several additional proteins were significantly altered in caspase-1, caspase-4 or NLRP3-knockdown cells (TGFα, CCL20, caspase-8, CSF-1, PDL-1, 4E-BP1, ADA and SCF), but these proteins currently lack a known association to the immune response during a UTI. The underlying mechanisms behind our observations are currently unknown but it may be primary or secondary effects. Inflammasome-associated proteins like NLRP3 have been shown to be transcriptional regulator of the innate immune response by facilitating e.g., the binding of IRF-4 to DNA^27^. Some of our observations may also be due to secondary phenomena i.e., a result of the reduced action of IL-1α and IL-1β, which are known to induce the expression of several other inflammatory proteins^15,23^. Taken together, the regulatory role of caspase-1, caspase-4 and NLRP3 on the inflammatory response in bladder epithelial cells seems to be broad, and not only associated with the processing of IL-1β and IL-18 to their active form.

A crucial part of the innate immune response to UPEC infection is the activation and migration of neutrophils to the urinary tract. Neutrophils are crucial for the clearance of UTI and they kill UPEC by phagocytosis, neutrophil extra cellular traps, ROS and the release of antimicrobial peptides^28^. As neutrophils are essential, we wanted to evaluate what role the caspase-1, caspase-4 and NLRP3 (knockdown) milieu from the bladder epithelium play in neutrophil migration, ROS-production and phagocytosis. We found that phagocytosis of CFT073 was increased in conditioned medium from UPEC-infected caspase-4-knockdown cells. In contrast, conditioned medium from UPEC-infected NLRP3-knockdown cells induced a significantly decreased phagocytosis of CFT073. Additionally, we also found that conditioned NLRP3-knockdown medium evoked significantly increased ROS-production from neutrophils. In contrast, conditioned medium from CFT073 stimulated caspase-4-knockdown cells induced a significantly decrease in ROS-production. As neutrophils are essential for the clearance of UPEC during UTI^28^, this phenotypical shift may have a biological effect on the outcome of the infection. Evaluating the proteomic data, we found that LIF was downregulated in conditioned medium from caspase-4-knockdown cells but upregulated by NLRP3- knockdown cells. LIF has been shown to reduce neutrophil phagocytosis^29^, which may explain the increased and decreased phagocytosis mediated by medium from caspase-4- and NLRP3-knockdown cells, respectively. The reduced phagocytosis may, theoretically, shift the antimicrobial phenotype of neutrophils to increased ROS production. We further evaluated the involvement of the inflammasome-associated proteins in neutrophil migration. However, we did not find any difference in neutrophil migration, which might be explained by the great interindividual variations we experienced in these experiments (data not shown). Furthermore, UPEC may suppress bladder epithelial NLRP3 by the virulence factor TcpC^16^ and thus be able to produce an NLRP3-deficent bladder milieu in UTI patient. Upon neutrophil arrival, the bladder milieu could promote a ROS-producing, rather than a phagocytic neutrophil phenotype. Taken together, we have shown that caspase-4 and NLRP3 are important modulators of the early host response during a UPEC infection. However, additional investigation is needed to further understand the role of caspase-4 in the outcome of UTI.

The regeneration and repair of the bladder epithelium is crucial during injury to maintain its function as a protective barrier against waste and cytotoxic products in the urine. A UPEC infection injures the highly differentiated superficial epithelial cell layer and exposes the underlying intermediate cells to bacteria which may facilitate formation of persistent UPEC reservoirs, associated with recurrent UTI, deeper into the urothelium^30^. We showed that conditioned medium from caspase-4 and NLRP3-knockdown cells induced an increased proliferation of wild-type bladder epithelial cells. If an active downregulation of inflammasome-associated proteins is induced by UPEC through e.g., TcpC^16^, the undifferentiated bladder epithelium would be exposed to a bladder milieu that would promote proliferation. Recently it was shown that during bladder epithelial injury the immune system prioritizes epithelial repair at the expense of UPEC clearance. This preference of the immune system was associated with recurrent UTI^31^. Hence, our findings show that caspase-4 and NLRP3 are involved in bladder epithelial proliferation and repair. However, further investigation is needed to understand how this contributes to the outcome of the infection.

In conclusion, our findings show that the contribution of caspase-1, caspase-4 and NLRP3 to the host–pathogen interaction during UTI is broad and involves cytokine and chemokine release and antimicrobial activities of neutrophils. Hence, we need more detailed understanding of which host response systems UPEC modulates to infect the host. With this knowledge, we will be able to better evaluate, prevent and treat this highly common infection.

## Supplementary Information

Supplementary Information.

## Data Availability

All data generated or analysed during this study are included in this published article (and its Supplementary Information files).
