# Supplementary material for: The role of caspase-1, caspase-4 and NLRP3 in regulating the host cell response evoked by uropathogenic Escherichia coli
Source: Sci Rep. 2022 Feb 7;12:2005. doi: 10.1038/s41598-022-06052-7 (PMC8821701; doi:10.1038/s41598-022-06052-7)
Supplement: Supplementary file 1 — Supplementary Information. [file 41598_2022_6052_MOESM1_ESM.pdf]

Supplementary information

The role of caspase-1, caspase-4 and NLRP3 in regulating the host cell response evoked by uropathogenic *Escherichia coli*

Anna Lindblad, Charlotte Johansson, Katarina Persson and Isak Demirel*

School of Medical Sciences, Inflammatory Response and Infection Susceptibility Centre (iRiSC), Örebro University, Örebro, Sweden

*Corresponding author: Isak Demirel, School of Medical Sciences, Campus USÖ, Örebro University, SE-701 82 Örebro, Sweden. *email:* isak.demirel@oru.se

Supplementary Table S1. Primers used for quantitative real-time PCR.

**Gene symbol**	**Oligonucleotide sequences (5´-3´)**
IL-1β	*F:* CCACAGACCTTCCAGGAGAATG<br>*R:* GTGCAGTTCAGTGATCGTACAGG
IL-18	*F:* GATAGCCAGCCTAGAGGTATGG<br>*R:* CCTTGATGTTATCAGGAGGATTCA
IL-1α	*F:* TGTATGTGACTGCCCAAGATGAAG<br>*R:* AGAGGAGGTTGGTCTCACTACC
IL-6	*F:* AGACAGCCACTCACCTCTTCAG<br>*R:* TTCTGCCAGTGCCTCTTTGCTG
IL-8	*F:* GAGAGTGATTGAGAGTGGACCAC<br>*R:* CACAACCCTCTGCACCCAGTTT
GAPDH	*F:* GTCTCCTCTGACTTCAACAGCG<br>*R:* ACCACCCTGTTGCTGTAGCCAA

**Supplementary Table S2.** Olink inflammation panel		
**Protein name**	**Abbreviation**	**Uniprot ID**
Eukaryotic translation initiation factor 4E-binding protein 1	4E-BP1	Q13541
Adenosine deaminase	ADA	P00813
Artemin	ARTN	Q5T4W7
Axin-1	AXIN1	O15169
Brain-derived neurotrophic factor	BDNF	P23560
Beta-nerve growth factor	Beta-NGF	P01138
Caspase 8	CASP-8	Q14790
C-C motif chemokine 11	CCL11	P51671
C-C motif chemokine 19	CCL19	Q99731
C-C motif chemokine 20	CCL20	P78556
C-C motif chemokine 23	CCL23	P55773
C-C motif chemokine 25	CCL25	O15444
C-C motif chemokine 28	CCL28	Q9NRJ3
C-C motif chemokine 3	CCL3	P10147
C-C motif chemokine 4	CCL4	P13236
Cluster of differentiation 244	CD244	Q9BZW8
Cluster of differentiation 40	CD40	P25942
Cluster of differentiation 5	CD5	P06127
Cluster of differentiation 6	CD6	Q8WWJ7
CUB domain-containing protein 1	CDCP1	Q9H5V8
Colony-stimulating factor 1	CSF-1	P09603
Cystatin D	CST5	P28325
Fractalkine	CX3CL1	P78423
C-X-C motif chemokine 1	CXCL1	P09341
C-X-C motif chemokine 10	CXCL10	P02778
C-X-C motif chemokine 11	CXCL11	O14625
C-X-C motif chemokine 5	CXCL5	P42830
C-X-C motif chemokine 6	CXCL6	P80162
C-X-C motif chemokine 9	CXCL9	Q07325
Delta and Notch-like epidermal growth factor-related receptor	DNER	Q8NFT8
Protein S100-A12	EN-RAGE	P80511
Fibroblasts growth factor 19	FGF-19	O95750
Fibroblasts growth factor 21	FGF-21	Q9NSA1
Fibroblasts growth factor 23	FGF-23	Q9GZV9
Fibroblasts growth factor 5	FGF-5	Q8NF90
Fms-related tyrosine kinase 3 ligand	Flt3L	P49771
Glial cell line-derived neurotrophic factor	GDNF	P39905
Hepatocyte growth factor	HGF	P14210
Interferon gamma	IFN-gamma	P01579
Interleukin-1 alpha	IL-1 alpha	P01583
Interleukin-10	IL10	P22301
Interleukin-10 receptor subunit alpha	IL-10RA	Q13651
Interleukin-10 receptor subunit beta	IL-10RB	Q08334

Interleukin-12 subunit beta	IL-12B	P29460
Interleukin-13	IL13	P35225
Interleukin-15 receptor subunit alpha	IL-15RA	Q13261
Interleukin-17A	IL-17A	Q16552
Interleukin-17C	IL-17C	Q9P0M4
Interleukin-18	IL18	Q14116
Interleukin-18 receptor 1	IL-18R1	Q13478
Interleukin-2	IL2	P60568
Interleukin-20	IL-20	Q9NYY1
Interleukin-20 receptor subunit alpha	IL-20RA	Q9UHF4
Interleukin-22 receptor subunit alpha-1	IL-22 RA1	Q8N6P7
Interleukin-24	IL-24	Q13007
Interleukin-2 receptor subunit beta	IL-2RB	P14784
Interleukin-33	IL33	O95760
Interleukin-4	IL4	P05112
Interleukin-5	IL5	P05113
Interleukin-6	IL6	P05231
Interleukin-7	IL7	P13232
Interleukin-8	IL8	P10145
Latency-associated peptide transforming growth factor beta-1	LAP TGF-beta-1	P01137
Leukemia inhibitory factor	LIF	P15018
Leukemia inhibitory factor receptor	LIF-R	P42702
Monocyte chemotactic protein 1	MCP-1	P13500
Monocyte chemotactic protein 2	MCP-2	P80075
Monocyte chemotactic protein 3	MCP-3	P80098
Monocyte chemotactic protein 3	MCP-4	Q99616
Matrix metalloproteinase-1	MMP-1	P03956
Matrix metalloproteinase-10	MMP-10	P09238
Neurturin	NRTN	Q99748
Neurotrophin-3	NT-3	P20783
Osteoprotegerin	OPG	O00300
Oncostatin-M	OSM	P13725
Programmed cell death 1 ligand 1	PD-L1	Q9NZQ7
Stem cell factor	SCF	P21583
SIR2-like protein 2	SIRT2	Q8IXJ6
Signaling lumphocytic activation molecule	SLAMF1	Q13291
Sulfotransferase 1A1	ST1A1	P50225
STAM-binding protein	STAMPB	O95630
Transforming growth factor alpha	TGF-alpha	P01135
Tumour necrosis factor	TNF	P01375
Tumour necrosis factor-beta	TNFB	P01374
Tumour necrosis factor receptor superfamily member 9	TNFRSF9	Q07011
Tumour necrosis factor ligand superfamily member 14	TNFSF14	O43557
TNF-related apoptosis-inducing ligand	TRAIL	P50591
TNF-related activation-inducing cytokine	TRANCE	O14788

Thymic stromal lymphopoietin	TSLP	Q969D9
Tumour necrosis factor (Ligand) superfamily, member 12	TWEAK	O43508
Urokinase-type plasminogen activator	uPA	P00749
Vascular endothelial growth factor A	VEGFA	P15692

Supplementary Figure S1

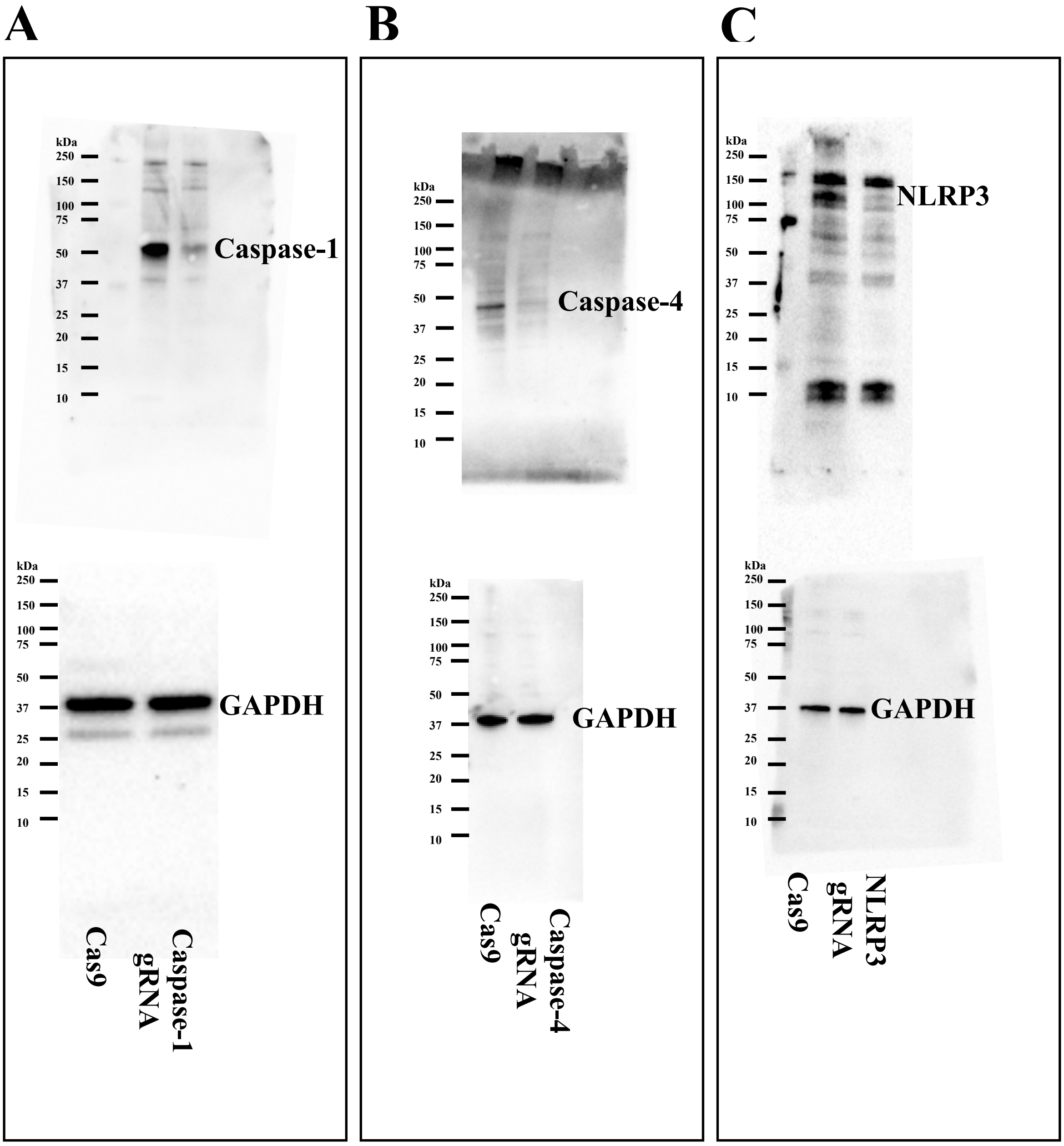

Supplementary Figure S1: Original whole western blots shown. The capsase-1 (A), caspase-4 (B), NLRP3 (C) and GAPDH (A-C) membranes were cut before antibody probing. This was done in order to probe for several proteins simultaneously.
